# Assessing Cell-to-Cell DNA Methylation Variability on Individual Long Reads

**DOI:** 10.1038/srep21317

**Published:** 2016-02-18

**Authors:** Wei Qu, Tatsuya Tsukahara, Ryohei Nakamura, Hideaki Yurino, Shin-ichi Hashimoto, Shoji Tsuji, Hiroyuki Takeda, Shinichi Morishita

**Affiliations:** 1 Department of Computational Biology and Medical Sciences, Graduate School of Frontier Sciences, the University of Tokyo, Kashiwa, Japan; 2 Department of Biological Sciences, Graduate School of Science, the University of Tokyo, Tokyo, Japan; 3 Graduate School of Medical Sciences, Kanazawa University, Kanazawa, Japan; 4 Department of Neurology, Graduate School of Medicine, the University of Tokyo, Tokyo, Japan; 5 Department of Neurobiology, Harvard Medical School, 220 Longwood Avenue, Boston, MA, USA; 6 CREST, JST, 5-3 Yonbancho, 4-1-8 Honcho, Kawaguchi, Saitama, Japan

## Abstract

Understanding cell-to-cell variability in cytosine methylation is essential for understanding cellular perturbation and its molecular machinery. However, conventional methylation studies have focused on the differences in the average levels between cell types while overlooking methylation heterogeneity within cell types. Little information has been uncovered using recent single-cell methods because of either technical limitations or the great labor required to process many single cells. Here, we report the highly efficient detection of cell-to-cell DNA methylation variability in liver tissue, based on comparing the methylation status of adjacent CpG sites on long sequencing reads. This method provides abundant methylation linkage information and enables genome-wide estimation of cell-to-cell variability. We observed repressed methylation variability in hypomethylated regions compared with the variability in hypomethylated regions across the genome, which we confirmed using public human sperm data. A gradual change in methylation status at the boundaries of hypomethylated regions was observed for the first time. This approach allows the concise, comprehensive assessment of cell-to-cell DNA methylation variability.

Although the DNA in a given cell population is identical, the epigenetic information in different cells, such as DNA methylation or histone modifications, is thought to differ. It is extremely important to understand cellular variability and its molecular machinery, although our current knowledge of cell-level epigenetic regulation remains poor. DNA methylation (5-methyl-cytosines at CpG sites) heterogeneity in a cell population has been observed recently using a single-cell sequencing approach^1^,^2^. However, there are difficulties in this strategy because DNA is invariably lost during various experimental steps, incomplete chemical reactions critically limit genome coverage, and its applicability to parallel analyses is limited when studying many cells. Conventional methods of profiling genome-wide DNA methylation, such as next-generation whole-genome bisulfite sequencing, have overlooked cell-to-cell variability and focused on the changes in methylation status between “average” cells at a single-base resolution (Fig. 1A)^3^,^4^.

An efficient alternative for detecting cell-to-cell methylation variability is to exploit adjacent CpG sites on individual reads. Reads are randomly sampled DNA molecules from a cell population, so the variability in their methylation patterns for a given locus stochastically reflects the variability in the cell population^5^. Compared with single-cell methylome studies, this approach cannot compare methylomes at the resolution of a single cell, but could provide the characteristics of cell-to-cell variability. Combining this with HiSeq sequencing, Landan *et al.* conducted a genome-wide epigenetic polymorphism study using the methylation patterns of four adjacent CpG sites on individual reads^6^. However, the genome coverage was extremely poor; only 0.3% of all CpG sites were covered and only CpG-rich regions were detectable due to the short length of the reads (36 bp). Here, we address these difficulties and limitations by using long MiSeq sequencing reads (2 × 300 bp paired-end reads), which can effectively and comprehensively observe the methylation status of 3 to 21 adjacent CpG sites on individual reads (Fig. 1A). In a moderately sequenced sample of the medaka, *Oryzias latipes* (12.7×), this strategy frequently covered (≥5×) 38.0% of all CpG sites when testing the methylation patterns of five adjacent CpG sites, or 8.4% when observing 11 adjacent CpG sites (Tables S1 & S2, See Methods). Using public HiSeq (100 bp reads) human sperm data^7^, we confirmed some of our findings, although the shorter read length limited the observation of linked methylation status. The sufficiency of genome coverage using longer reads allows a deeper understanding of cell-to-cell DNA methylation variability.

## Results

### Obtaining linked DNA methylation on long reads

We performed whole-genome bisulfite sequencing (WGBS) of adult liver tissue from the medaka, *Oryzias latipes* (Hd-rR strain), because it consists mostly of hepatocytes and is relatively homogeneous (See Methods). Compared with the human genome, the medaka genome has a higher density of CpG sites. The median distance between adjacent CpG sites is 24 bases in the medaka genome versus 41 bases in the human genome. We used MiSeq v2 and MiSeq v3 paired-end sequencing and obtained 34.2 million reads of 2 × 250 bp or 2 × 300 bp (Table S1). After removing poor-quality reads and those with a higher risk of experimental redundancy or imperfect bisulfite conversion^8^, 20.3 million reads were uniquely mapped to give a genome coverage of 12.7× (See Methods). The read coverage at individual CpG sites was approximately normally distributed, which indicated the unbiasedness and sufficiency of our experimental method (Fig. S1). The methylation rate of non-CpG cytosines was as low as 1.2% for MiSeq sequencing and 1.3% for Sanger sequencing using TA cloning experiments, as in our previous study^9^, which suggested sufficient bisulfite conversion. Overall, 97.9% of the CpG sites in the medaka genome were covered and 88.3% remained for further analysis. CpG sites with poor coverage (fewer than five reads) or located in repeat regions that covered more than 30 reads were discarded.

### Repressed methylation variability in hypomethylated regions

**Figure 1:**
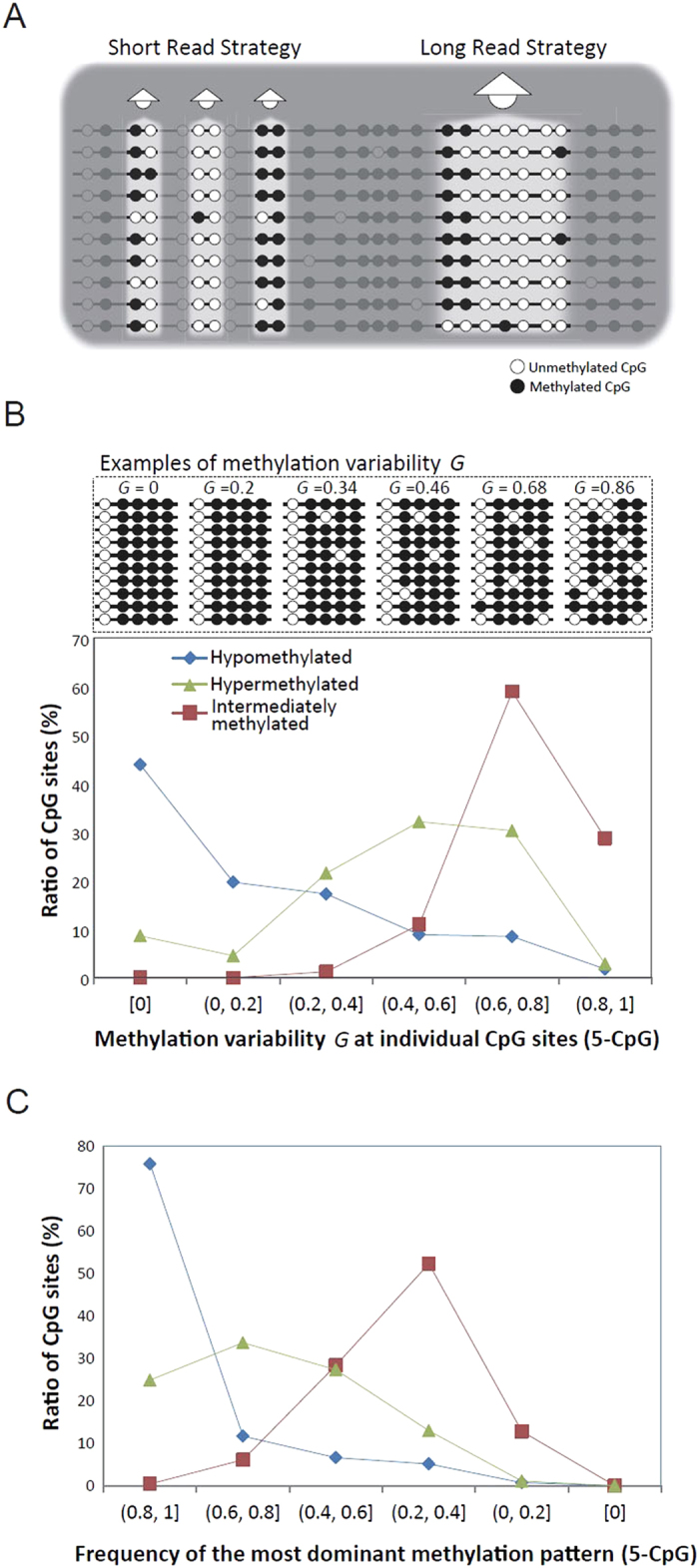
The DNA Methylation variability, *G*, on long individual reads. (**A**) Different degrees of methylation linkage can be observed on conventional short reads versus long reads. (**B**,**C**) The distributions of cell-to-cell methylation variability (adjacent five CpG sites) vary significantly among hyper, hypo-, and intermediately methylated CpG sites, as do the distributions of the frequency of the most dominate methylation pattern among them.

To address the essential question regarding cell-to-cell variability, *i*.*e*., how much does the cytosine methylation status vary throughout a cell population?, we analyzed the pattern and distribution of adjacent CpG sites on individual reads. We observed genome-wide methylation patterns of 3 to 21 adjacent CpG sites (Table S2). The methylation variability at a specific CpG site was measured using the Gini index, ***G*** = 1 − Σ(*P*_*i*_^2^), where *P*_*i*_ is the probability of methylation pattern *i* being observed on individual reads. When all of the patterns are the same, ***G*** = 0; when the patterns vary markedly, ***G*** is close to 1. Figure 1B shows the distribution of the variability of ***G*** for hyper-, hypo-, and intermediately methylated CpG sites, using the methylation patterns of five adjacent CpG sites. Interestingly, the methylation variability was much smaller at hypomethylated CpG sites across the genome. When a CpG site is hypomethylated, 43.9% of the five CpG methylation patterns have zero variability (***G*** = 0, all five adjacent CpG sites are unmethylated) and 10.2% have high variability (***G*** ≥ 0.6). Conversely, when a CpG site is hypermethylated, only 8.6% have zero variability (***G*** = 0, all five adjacent CpG sites are methylated) and 33.1% have high variability (***G*** ≥ 0.6). Correspondingly, the distributions of the frequency of the most dominant methylation pattern vary vastly among hyper- and hypo-methylated CpG sites (Fig. 1C and Fig. S3). A similar tendency was observed in all 3 to 21 adjacent CpG methylation patterns (Fig. S4). In addition, intermediately methylated CpG sites had the highest variance in methylation patterns, albeit trivially. We confirmed that longer CpG fragments have higher variability in ***G*** (Fig. S4). These results suggest that cell-to-cell methylation dynamics are stochastic; the degree is significant, and it varies according to the distinct methylation status. Hypomethylated regions have much smaller cell-to-cell methylation variability than do hypermethylated regions.

To confirm this finding, we examined cell-to-cell methylation variability using publicly available human sperm data from two donors^7^. These data were generated using bisulfite and HiSeq (100 bp single-end and paired-end reads). We mapped reads using bsmap^10^ and removed duplicates with the same start positions (See Methods). We obtained substantial numbers of CpG sites when testing the methylation patterns of the five adjacent CpG sites, 3.2% (1,001k) and 2.3% (701k) of all of the CpG sites for the two donors, respectively. The distribution of methylation variability (***G***) of hyper-, hypo-, and intermediately methylated CpG sites in the human sperm data was similar to what we obtained from longer reads of the medaka liver data. That is, hypomethylated CpG sites have much smaller methylation variability (Fig. S5). We also examined methylation variability (***G***) using public single-cell methylation data from 12 mouse oocytes^2^. Single-cell bisulfite sequencing data provided the degree of methylation of adjacent CpG sites in a single cell, allowing direct comparisons of the patterns among cells. In total, we obtained 0.3% (105k sites) of the CpG sites when testing the methylation patterns of five adjacent CpG sites (See Methods). These data were less sufficient. Note that adjacent hypermethylated CpG sites were poorly covered (only 124 CpG sites in 105k). In conclusion, we obtained approximately similar distributions of methylation variability (Fig. S6).

As there are two DNA molecules per diploid cell, and each DNA molecule has two strands, this generates four individual reads in total from a single region of the genome. The variability that we observed on long individual reads might originate within the four fragments of a single cell, but the possibility is extremely low because of the inevitable huge loss of DNA during the various experimental steps and because the reads were sampled randomly from a large pool of DNA molecules. Our WGBS experiment started with 1~3 μg of DNA, but less than 100 *p*g of DNA were ultimately sequenced. Furthermore, we trimmed PCR and sequencing replicates (less than 5% of the reads) by removing reads that were mapped to a same start location on the reference. These procedures significantly lowered the rate of collision, *i*.*e*., the rate of reads covering the same CpG site coming from the same cell. Therefore, most of the variability observed among reads can be considered differences in cell-to-cell DNA molecules.

The observed cell-to-cell methylation variability could be caused by unfaithful maintenance of DNA methylation or *de novo* methylation. Regardless of the high accuracy of DNA methyltransferase 1 (Dnmt1), studies using hairpin-bisulfite to examine DNA methylation fidelity have revealed a much lower methylation inheritance in hypermethylated CpG islands (83%) compared with that in hypomethylated CpG islands (94–99%)^11^,^12^,^13^. If we assume that this inaccurate methylation transmission happens at every cell division, after roughly five divisions only half of the original methylation information remains in the daughter cells. In fact, actual *in vivo* methylation transmission is more complicated^14^. Another recent, comprehensive hairpin-bisulfite experiment found a correlation between methylation fidelity and gene expression^15^. We further compared the methylation variability with our previously published nucleosome occupancy around transcription state sites (TSS) and gene bodies (Fig. S7)^16^, and found that this correlation could be an effect of the relationship between gene expression level and the degree of methylation. The greater the expression of a gene is, the lower the degree of methylation at the TSS. Instead of observing single-cell-based methylation maintenance for a limited genomic region, using long reads enabled the efficient detection of the linkage of methylation patterns and their genome-wide variability in a cell population.

## Methylation variability at the boundaries of hypomethylated regions

**Figure 2:**
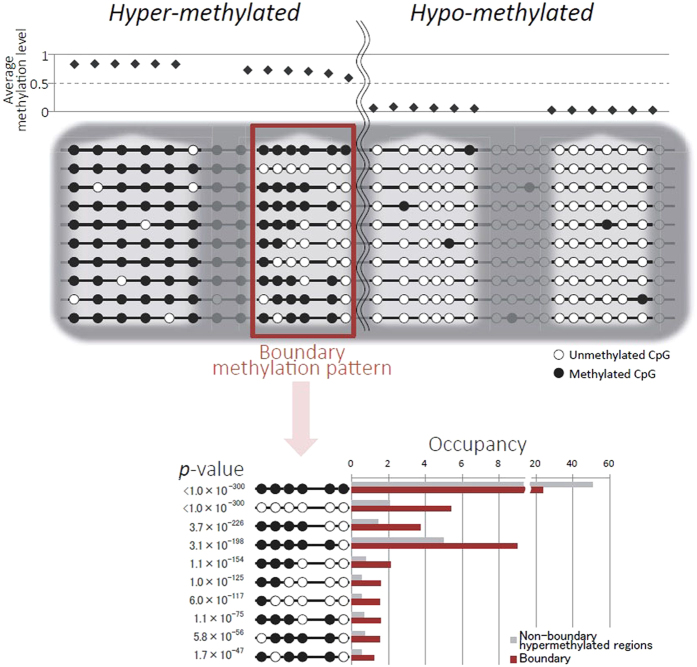
Gradual change in the methylation status at reliable boundaries of hyper- and hypomethylated regions. The conventional detection of DNA methylation shows the average methylation level on the top. Our detection using long reads presents the details of various methylation patterns in non-boundary hypermethylated regions, reliable boundaries of hyper- and hypomethylated regions, and inside hypomethylated regions. Specifically, the top ten patterns in the two proportion *z*-test between boundary patterns (occupancy ≥1%) and those in non-boundary hypermethylated regions are listed on the bottom.

As another example, it is intriguing to ask how the methylation pattern changes near the boundaries of hypomethylated regions. Does the status change abruptly or gradually at the boundary? The individual long reads used in our study show a clear gradual change in a cell population (Fig. 2). Unlike conventional methylation studies, which demonstrate a sharp change in the average methylation level (from 0.59 to 0.05) at the boundary between hyper- and hypomethylated regions, adjacent CpG methylation observations provide much more detail. Two proportion z-tests were run between boundary patterns (occupancy ≥1%) and those in non-boundary hypermethylated regions. The ten patterns with the most markedly different proportions are listed at the bottom of Fig. 2. The top seven patterns support gradual changes at the boundary and these are specifically located outside the hypomethylated regions. In comparison, no similar gradual change was detected immediately inside the hypomethylated region boundaries (Fig. S8, See Methods). We performed the same test on hypomethylated region boundaries using less strict criteria and obtained very similar results (Fig. S9, See Methods). These observations reveal the detailed cell-to-cell methylation dynamics in the formation of hypomethylated regions. The gradual patterns at these boundaries suggest dynamic variability when establishing proximal hypermethylated regions, which may arise from three-dimensional chromatin transformation during cell development.

## Discussion

In this study, we present an efficient, informative method for analyzing cell-to-cell DNA methylation patterns in liver tissues by mining adjacent CpG sites on individual long reads. This approach is unable to compare methylomes at a “single-cell” resolution, but provided fundamental characteristics of cell-to-cell methylation variability. Across the entire genome, methylation variability is repressed in hypomethylated regions. Immediately outside these regions there is a gradual change in the methylation status in a cell population. If cell-to-cell methylation variability is caused by the unfaithful maintenance of DNA methylation, then this variability would be an aging-related or cell-division-related phenomenon. A study found that the interindividual variation in twins with regard to locus-specific DNA methylation was greater in old twins than in young twins^17^.

We were concerned whether the bisulfite conversion efficiency (98.8%, if we suppose the 1.2% non-CpG cytosines observed as methylated were all unmethylated) would affect the evaluation of methylation variability. Incomplete bisulfite conversion will result in an unmethylated cytosine being detected as a methylated one, which will increase the variability in hypomethylated regions rather than in hypermethylated regions. This will not affect our finding regarding repressed variability in hypomethylated regions.

Another concern is that liver tissue is not solely hepatocytes, but a mixture containing significant proportions of different types of cells, such as sinusoidal endothelial cells and hepatic stellate cells^18^. This could affect the robustness of the variation in cell-to-cell methylation observed herein. However, available human sperm data using shorter reads supported our finding regarding repressed methylation variability in hypomethylated regions. Further analyses using this method, such as comparing different cell samples, should reveal more details about epigenetic cellular variability.

## Materials and Methods

### Construction and sequencing of bisulfite-treated DNA libraries

Bisulfite-treated DNA libraries of medaka tissue (liver from the Hd-rR strain) were constructed as follows. Genomic DNA from medaka tissue cells was isolated according to the manufacturer’s protocol using the DNeasy Blood & Tissue Kit (QIAGEN, Germantown, MD, USA). DNA concentration and purity were determined by optical density measurements using a NanoDrop spectrophotometer (BioTek Instruments, Winooski, VT, USA). To obtain fragmented DNA of the desired size range (500–800 bp), 7 μg of genomic DNA was sonicated for 80 seconds using a Covaris sonicator. The DNA fragments were treated with Klenow DNA polymerase to generate blunt ends and were ligated with double-stranded DNA adaptors containing methylated cytosines, which were designed to amplify only those DNA fragments with bisulfite-converted adaptor sequences at both ends. The sequences of these methylation linkers were:

5′-C^m^C^m^AC^m^CAC^m^GC^m^C^m^CC^m^C^m^GC^m^CCTC^m^C^m^CC^m^TC^m^CACGGGC^m^AGTC^m^GGTGAT-3′, 5′-ATCACCGACTGCCCGTGGAGGGGAGGGCGGGGGCGTGGTGGTT-3′, 5′-GACGCCCAAGAGAACGAGGAAC^m^C^m^C^m^G-3′, and 5′-TTTCGGGTTCCTCGTTCTCTTGGGCGTC-3′.

DNA fragments were purified using 30–50 μL AMPure XP beads (Beckman-Coulter, Brea, CA, USA), and eluted off the beads in 20 μL of distilled water. Bisulfite was modified using an EpiTect Bisulfite Kit (QIAGEN), followed by PCR amplification using EpiTaq™ HS (Takara Bio, Shiga, Japan). The reaction conditions were an initial denaturation at 94 °C for 5 minutes; 7–10 cycles of 98 °C for 10 s, 55 °C for 30 s, and 72 °C for 60 s; and a final 5-minute extension at 72 °C. The following primer sequences were used: converted linker-specific primer A (P5 long F primer) 5′-AATGATACGGCGACCACCGAGATCTACACCCACTACGCCTCCGC TTTCCTCTCTATGG-3′, converted linker-specific primer B (P7 long R primer) 5′-CAAGCAGAAGACGGCATACGAGATCGGGTTCCTCATTCTCTTAAACATC-3′, FDV Read 1 Seq Primer 5′-CCTCCGCTTTCCTCTCTATGGGCAGTCGGTGAT-3′, and RDV Read 2 Seq Primer 5′-GGCATACGAGATCGGGTTCCTCATTCTCTTA AACATC-3′, which were designed to amplify only those DNA fragments with bisulfite-converted adaptor sequences at both ends. The PCR products were size-fractionated by 4% polyacrylamide gel electrophoresis, and those 600–800 bp in length were recovered. Double-stranded libraries were quality checked on a High Sensitivity DNA Agilent chip run on the Agilent 2100 Bioanalyzer (Agilent Technologies) for size and molarity determination. The size-fractionated cDNAs were sequenced using an Illumina MiSeq system. The bisulfite conversion rate was estimated from a validation experiment, which followed a procedure similar to that using genomic DNA from *S*. *cerevisiae* S288C^9^.

### Mapping and processing reads

A read pair was 500–600 bp in length. To map a bisulfite-treated read pair to the reference genome efficiently, we converted all of the cytosines on the left read into thymines, and converted the guanines on the right read into adenines and then used the reverse complement sequence for the alignment. BWA-MEM was used to map a read pair^8^, followed by the following filters: the interval of two arms of a read pair was 100–800 bp, two arms of a read pair were uniquely mated on the genome, MAPQ ≥30. In addition, read pairs mapped to the same start location on the reference were removed to reduce PCR and sequencing redundancy. Smith–Waterman alignments between these reads and the original genome were used to evaluate the match rate and final methylation status. To avoid overestimating the methylation variability, read pairs were discarded if they had a low sequencing quality value (average QV <20), or >3% methylated non-CpG or >4% unmatched bases. CpG sites likely to be located in repeat regions (covered by more than 30 reads) or poorly covered (fewer than five reads) were also discarded. For mapping HiSeq human sperm reads, we performed single-end read mapping using bsmap with the same criteria.

### Calculating methylation variability

The methylation variability at a particular CpG site was measured using the Gini index, ***G*** = 1 − Σ(*P*_*i*_^2^), where *P*_*i*_ is the probability of methylation pattern *i* being observed on individual paired-end reads. We compared ***G*** for CpG sites of certain lengths (3~21). A ***G*** of length 5 CpG sites means that there was methylation variability at a specific CpG site ±2 CpG sites. If the occurrence of these five CpG sites on individual reads was low (less than five), the methylation variability at this particular CpG site was discarded. Obviously, if the methylation patterns around a particular CpG site were the same, ***G*** = 0 and the maximum *P*_*i*_, then the probability of the most dominant methylation pattern was 1. Conversely, if the methylation patterns around a CpG site varied substantially, ***G*** would be close to 1 and the maximum *P*_*i*_ would be smaller. Details of the methods can be found in the Supplemental Methods.

We also calculated methylation variability using public single-cell methylation data from 12 mouse oocytes. Methylation pattern *i* was observed in single cells and the methylation variability was discarded when fewer than five cells were available.

### Estimation of hypomethylated regions

The level of methylation of a particular cytosine was estimated by dividing the number of mapped reads reporting a cytosine (C) by the total number of reads reporting a C or T (thymine). A hypomethylated region was defined as when at least three consecutive CpG sites were hypomethylated (methylation level ≤0.2) and no more than two consecutive CpG sites had methylation levels ≥0.5. The boundary of the hypomethylated region starts and ends at a hypomethylated CpG site. Reliable hypomethylated region boundaries in further methylation variability analyses were strictly limited to long hypomethylated regions with ≥20 CpG sites, and many neighboring CpG sites around their boundaries (<6 CpG sites within ±10 CpG sites have an invalid ***G*** of length of 5 CpG sites).

### Testing specific methylation patterns on hypomethylated boundaries

We used two proportion *z*-tests and applied the Bonferroni correction to the ratio of all methylation patterns between the boundaries of hypomethylated regions and non-boundaries regions. Two comparisons were performed: immediately outside the hypomethylated region boundaries, compared with the entire genome, but excluding hypomethylated regions and 10 CpG sites proximal to the boundaries, and immediately inside the reliable hypomethylated region boundaries, compared with reliable hypomethylated regions but excluding the 10 CpG sites proximal to their boundaries (Fig. 2). We performed the same test on hypomethylated region boundaries using less strict criteria (Fig. S9).

## Additional Information

**Data Availability**: All sequence data are deposited at the NCBI BioProject PRJNA279817 (http://www.ncbi.nlm.nih.gov/bioproject/279817).

**How to cite this article**: Qu, W. *et al.* Assessing Cell-to-Cell DNA Methylation Variability on Individual Long Reads. *Sci. Rep.* **6**, 21317; doi: 10.1038/srep21317 (2016).

## Supplementary Material

Supplementary Information
