# Supplementary material for: Assessing Cell-to-Cell DNA Methylation Variability on Individual Long Reads
Source: Sci Rep. 2016 Feb 18;6:21317. doi: 10.1038/srep21317 (PMC4758075; doi:10.1038/srep21317)
Supplement: Supplementary Information [file srep21317-s1.pdf]

Supplementary Information

Assessing Cell-to-Cell DNA Methylation Variability on Individual Long Reads

Wei Qu[1,6#], Tatsuya Tsukahara[2,5], Ryohei Nakamura[2,6], Hideaki Yurino[3], Shin-ichi Hashimoto[3], Shoji Tsuji[4], Hiroyuki Takeda[2,6], Shinichi Morishita[1,6#]

[1] Department of Computational Biology and Medical Sciences, Graduate School of Frontier Sciences, the University of Tokyo, Kashiwa, Japan

[2] Department of Biological Sciences, Graduate School of Science, the University of Tokyo, Tokyo, Japan

[3] Graduate School of Medical Sciences, Kanazawa University, Kanazawa, Japan

[4] Department of Neurology, Graduate School of Medicine, the University of Tokyo, Tokyo, Japan

[5] Department of Neurobiology, Harvard Medical School, 220 Longwood Avenue, Boston, MA, USA

[6] CREST, JST, 5-3 Yonbancho, 4-1-8 Honcho, Kawaguchi, Saitama, Japan

[#] Correspondence should be addressed to W.Q. (quwei@cb.k.u-tokyo.ac.jp) and S.M (moris@cb.k.u-tokyo.ac.jp)

Supplementary Figure 1.

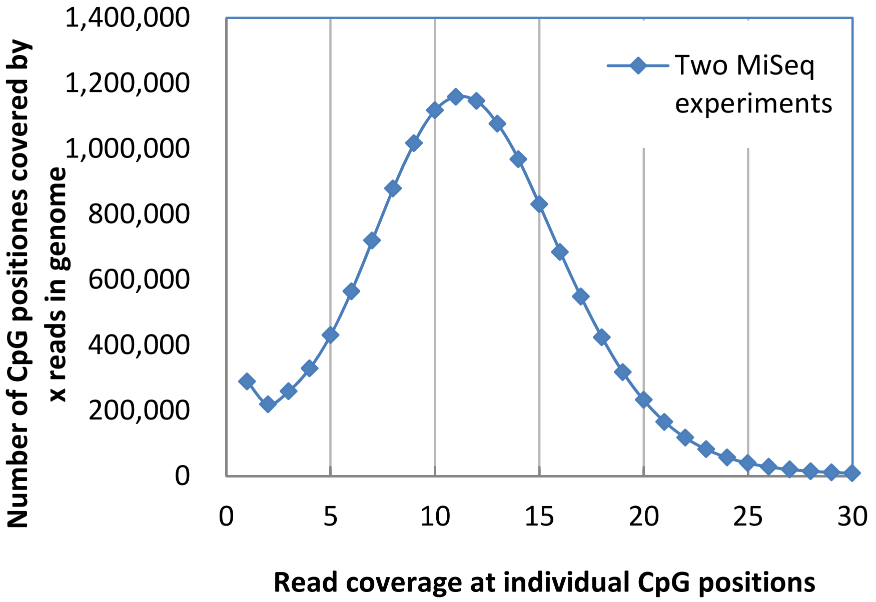

Supplementary Figure 2.

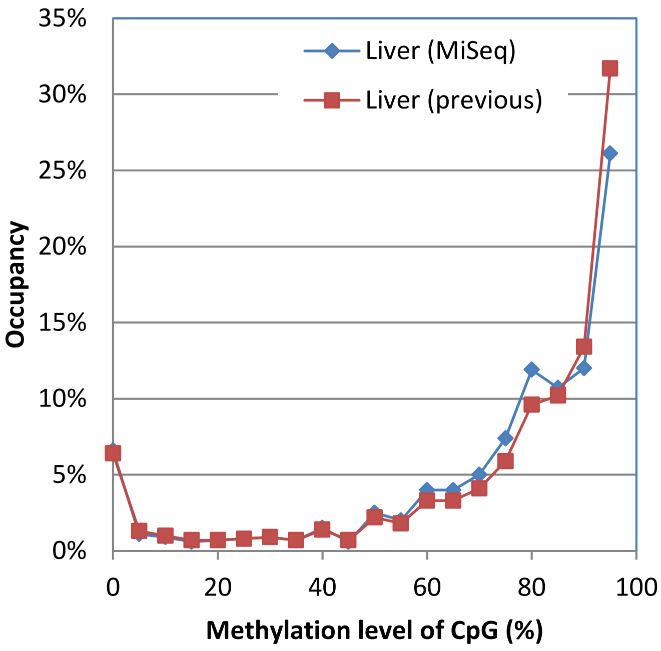

Supplementary Figure 3.

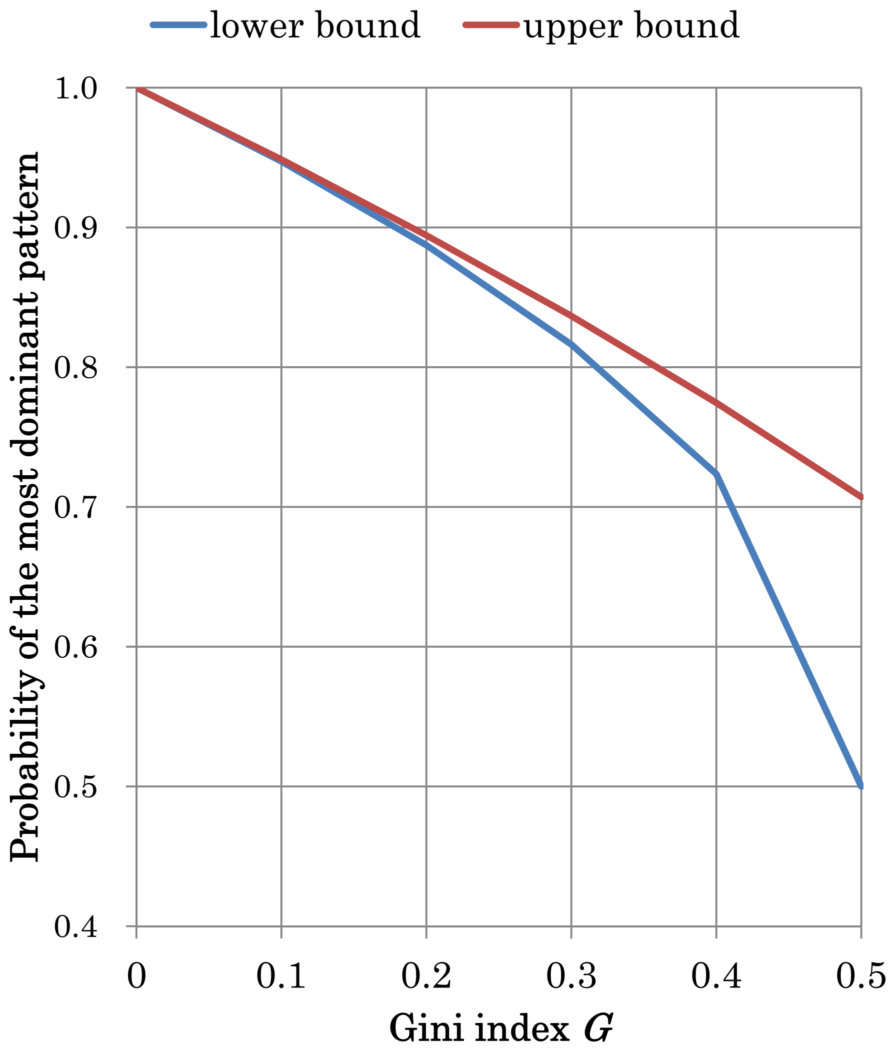

Supplementary Figure 4.

A.

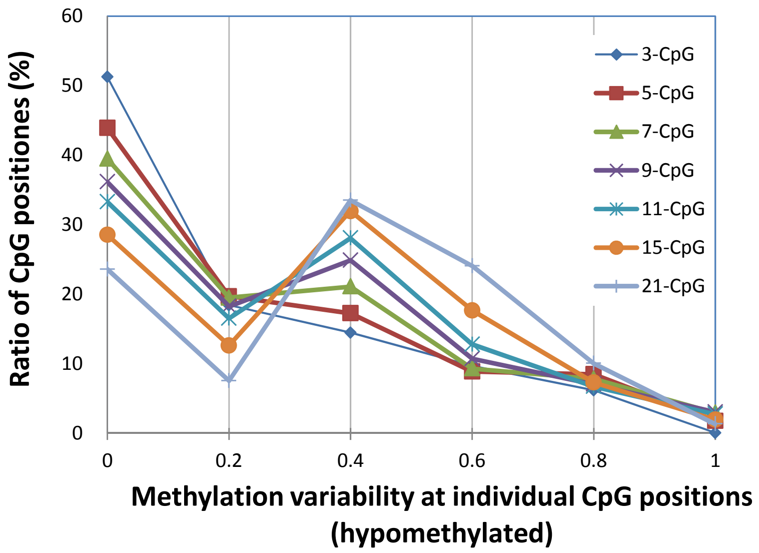

B.

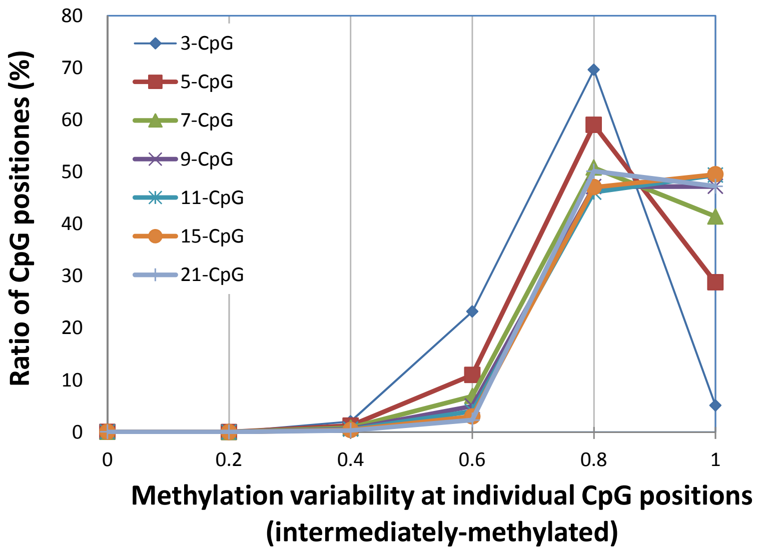

C.

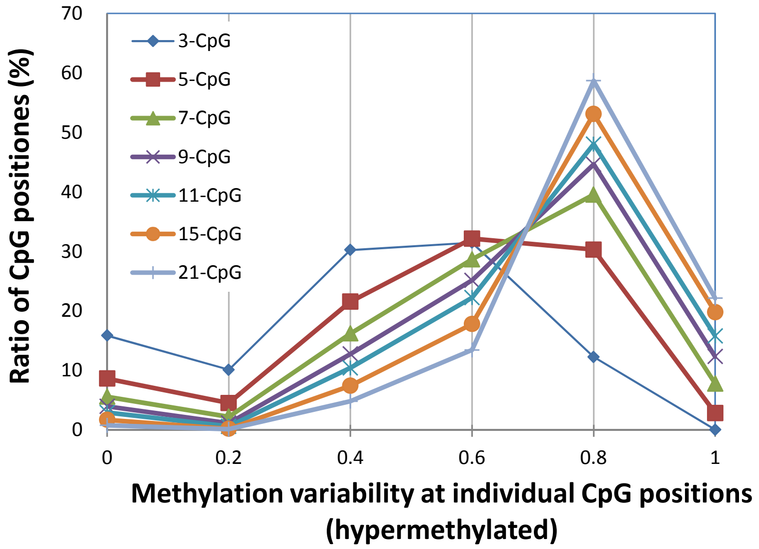
70
60
50
40
30
20
10
0
3-CpG
5-CpG
7-CpG
9-CpG
11-CpG
15-CpG
21-CpG
Ratio of CpG positiones (%)
0
0.2
0.4
0.6
0.8
1
Methylation variability at individual CpG positions
(hypermethylated)

Supplementary Figure 5.

A.

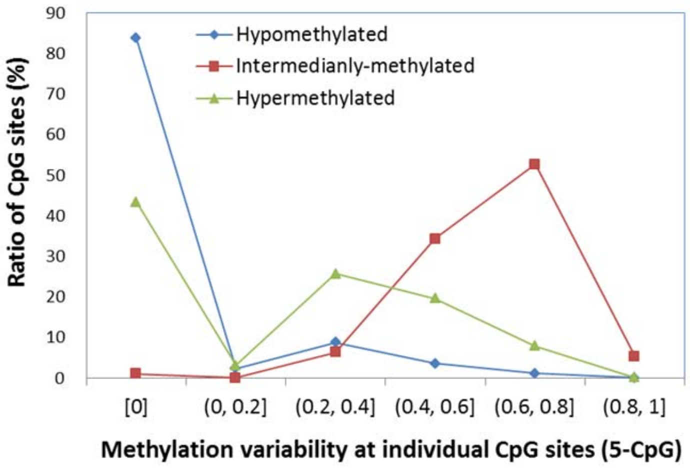

B.

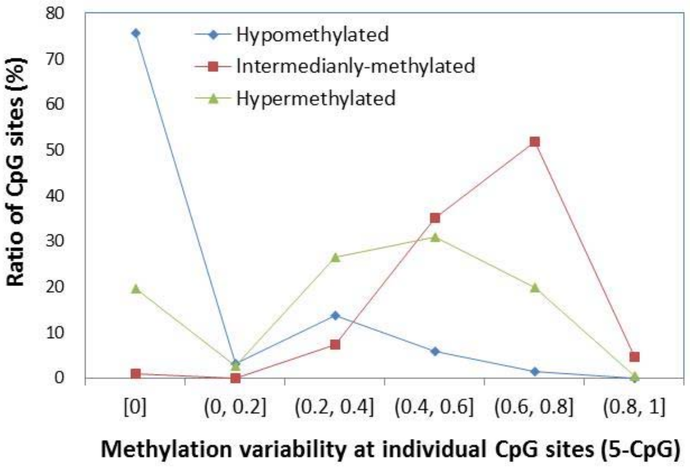

C.

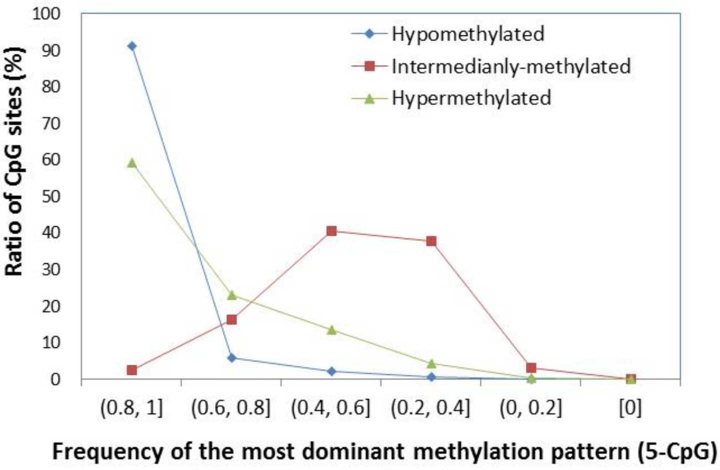

100
90
80
70
60
50
40
30
20
10
0
Ratio of CpG sites (%)
Hypomethylated
Intermedianly-methylated
Hypermethylated
(0.8, 1]
(0.6, 0.8]
(0.4, 0.6]
(0.2, 0.4]
(0, 0.2]
[0]
Frequency of the most dominant methylation pattern (5-CpG)

D.

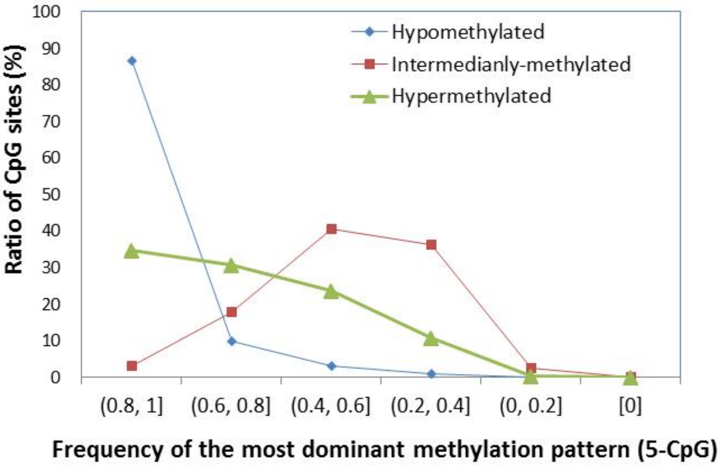

100
90
80
70
60
50
40
30
20
10
0
Ratio of CpG sites (%)
Hypomethylated
Intermedianly-methylated
Hypermethylated
(0.8, 1]
(0.6, 0.8]
(0.4, 0.6]
(0.2, 0.4]
(0, 0.2]
[0]
Frequency of the most dominant methylation pattern (5-CpG)

Supplementary Figure 6.

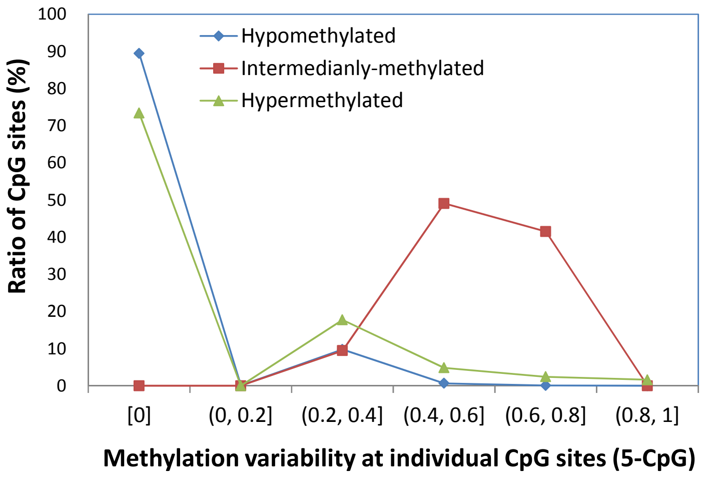

Supplementary Figure 7.

A.

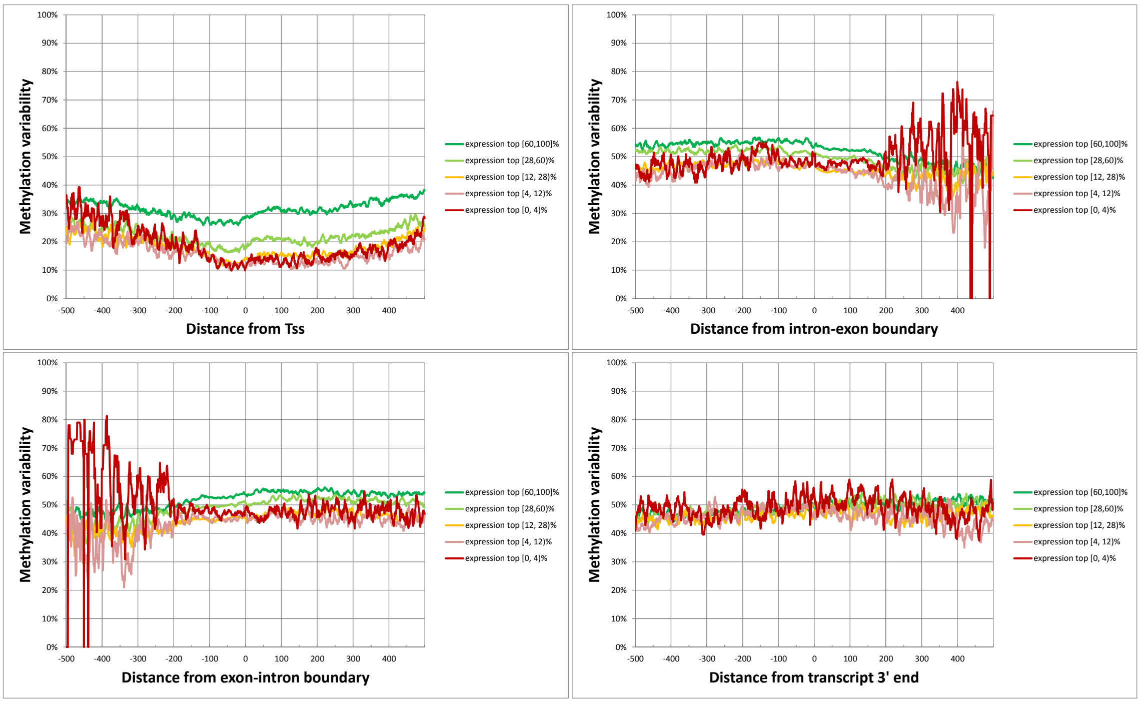

B.

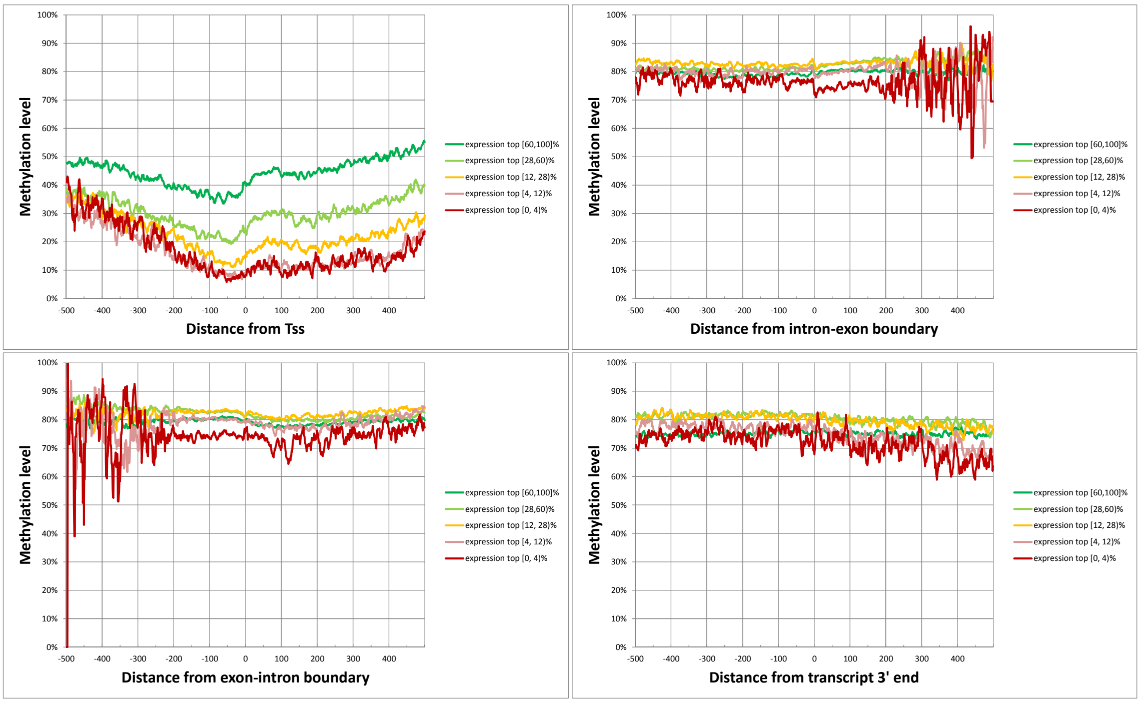

C.

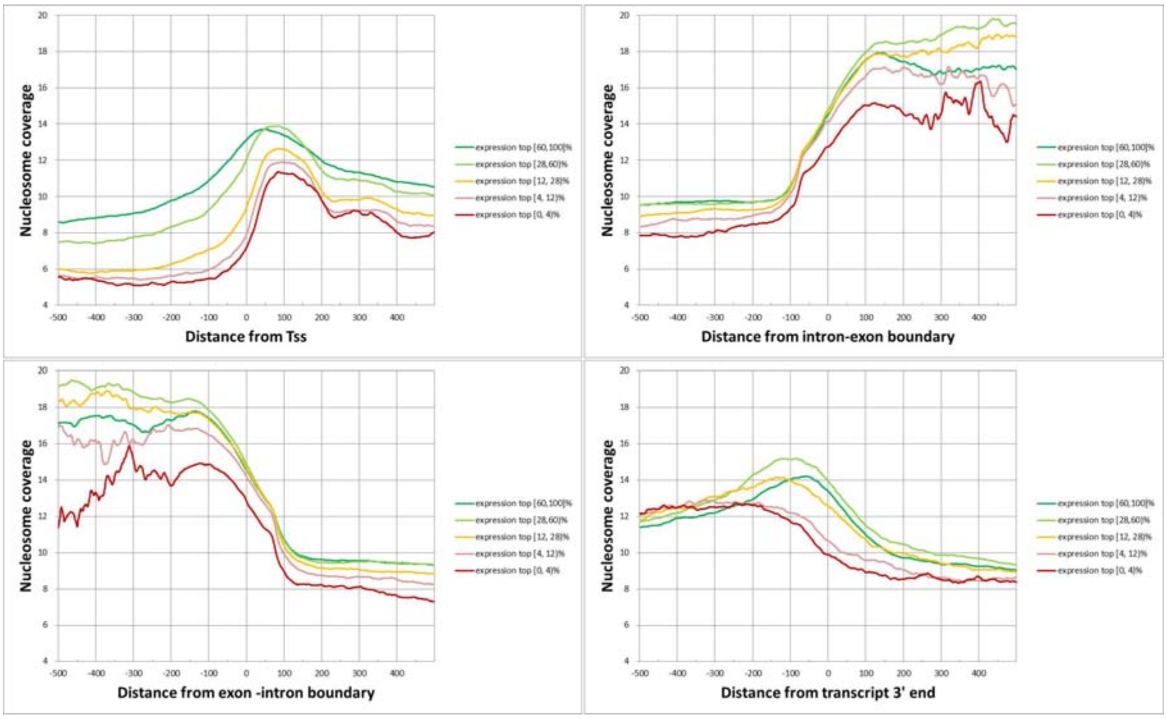

Supplementary Figure 8.

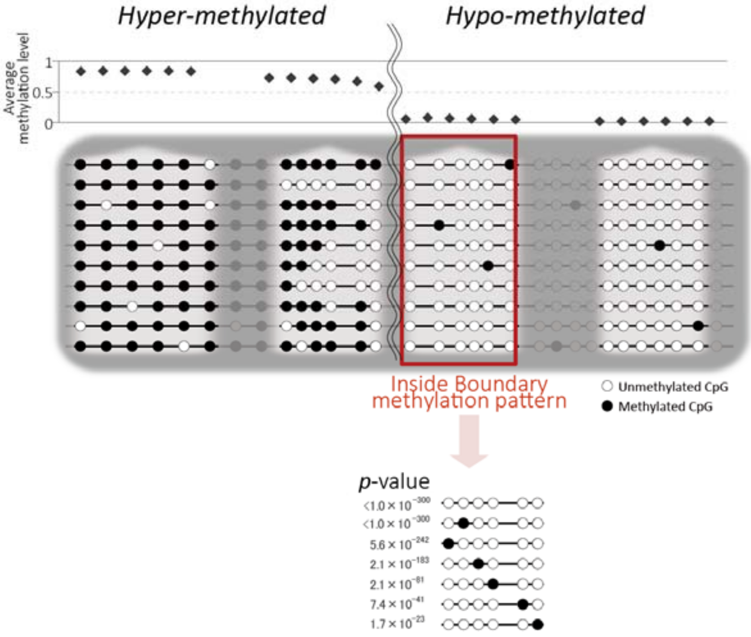

Supplementary Figure 9.

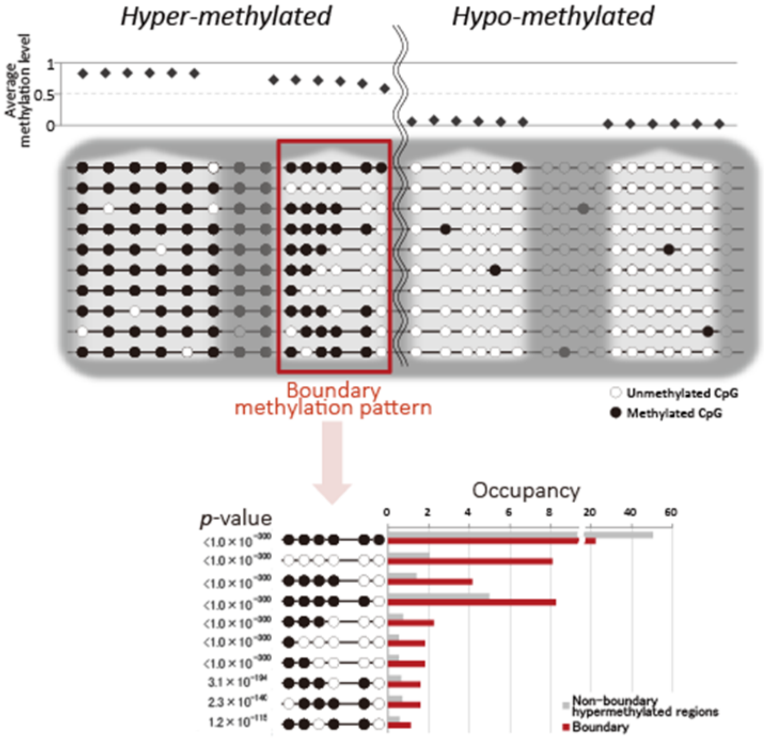

Supplementary Figure Legends

Supplementary Figure 1. Distribution of read coverage on individual CpG sites.

Supplementary Figure 2. Distribution of methylation level of individual CpG sites. The results of MiSeq (blue) method are highly concordant with results of previous HiSeq (red) method.

Supplementary Figure 3. Correspondence between methylation variability G and frequency of the most dominant methylation pattern**.**

Let α denote a string of letters 0 and 1 of length n, where 0 and 1 respectively indicate unmethylated and methylated CpGs. We call α a CpG methylation pattern. For example, 00110 indicates a CpG methylation pattern of five CpG sites such that the third and fourth CpGs are methylated while the other CpGs are unmethylated.

Let S denote a set of all CpG methylation patterns of length n. The number of CpG methylation patterns in S is 2^n. Suppose that $P(\alpha)$ denote the probability that pattern α occurs in reads that cover at least n CpG sites surrounding a focal CpG site. Note that $\sum_{i=1,\ldots,2^n} P(\alpha_i) = 1$, where we assume that $P(\alpha_1) \geq P(\alpha_2) \geq \cdots \geq P(\alpha_{2^n}) \geq 0$ without loss of generality. We call α_1 the most dominant pattern in S.

To measure the variety of CpG methylation patterns, we define the Gini index, $G = 1 - \sum_{i=1,\ldots,2^n} P(\alpha_i)^2$. The following property links the Gini index and the probability of the most dominant pattern.

$$\sqrt{\frac{1}{2}\left(\frac{1}{2}-G\right)}+\frac{1}{2} \le P(\alpha_1) \le \sqrt{1-G}.$$

To prove the left inequality, observe

$$1-G = \sum_{i=1,\dots,2^n} P(\alpha_i)^2$$

$$= P(\alpha_1)^2 + \sum_{i=2,\dots,2^n} P(\alpha_i)^2 \le P(\alpha_1)^2 + \left(\sum_{i=2,\dots,2^n} P(\alpha_i)\right)^2$$

$$= P(\alpha_1)^2 + \left(1-P(\alpha_1)\right)^2 = 2\left(P(\alpha_1)-\frac{1}{2}\right)^2 + \frac{1}{2}$$

It is easy to see $\sqrt{\frac{1}{2}\left(\frac{1}{2}-G\right)}+\frac{1}{2} \le P(\alpha_1)$.

To derive the right inequality, observe:

$$G-1= \sum_{i=1,\dots,2^n} P(\alpha_i)^2 = P(\alpha_1)^2 + \sum_{i=2,\dots,2^n} P(\alpha_i)^2 \ge P(\alpha_1)^2$$

Supplementary Figure 3 illustrates that the probability of the most dominant pattern is quite high when the Gini index gets smaller. For example, $0.724 \le P(\alpha_1)$, when $G \le 0.4$.

Supplementary Figure 4. DNA Methylation variability *G* on 3 to 21 coexisting CpG methylation patterns. (**A**) hypomethylated regions. (**B**) intermediately-methylated regions. (**C**) hypermethylated regions.

Supplementary Figure 5. DNA Methylation variability ***G*** on publicly available human sperm individual HiSeq reads (100bp). **(A)** and **(B)**, distributions of cell-to-cell methylation variability (adjacent five adjacent CpG sites) significantly vary among hyper, hypo- and intermediately-methylated CpG sites of donor#1 and donor#2, respectively. **(C)** and **(D)**, so do distributions of frequency of the most dominate methylation pattern in among them.

Supplementary Figure 6. Distributions of cell-to-cell methylation variability (adjacent five adjacent CpG sites in a single cell) on publicly available single-cell methylation data among twelve mouse oocytes.

Supplementary Figure 7. Methylation variability *G* (**A**), methylation level (**B**) and nucleosome occupancy (**C**) around transcription state sites and gene bodies.

Supplementary Figure 8. Gradual change in the methylation status at reliable boundaries of hyper- and hypomethylated regions. For immediately inside of hypomethylated region boundaries, the top ten patterns in the two proportion z-test between boundary patterns (occupancy $\geq$ 1%) and those in non-boundary hypermethylated regions are listed on the bottom.

Supplementary Figure 9. Gradual change in the methylation status at boundaries of hyper- and hypomethylated regions detected by less strict criteria. The top ten patterns in the two proportion z-test between boundary patterns (occupancy $\geq$ 1%) and those in non-boundary hypermethylated regions are listed on the bottom.

Supplementary Table 1. Sequencing and mapping summary.

Sample	Number of raw reads	Number of mapped read	Number of uniquely mapped	Genome coverage	Ratio of CpG covered by > 1
Experiment	14,602,846	10,088,447	9,616,866	6.0	-
Experiment	19,635,575	11,292,026	10,696,531	6.7	-
Sum of two MiSeq experiments	34,238,421	21,380,473	20,313,397	12.7	97.9%

Supplementary Table 2. Coverage of co-existing CpGs on individual reads.
A CpG is counted when its occurrence > 5.

Length of co-existing CpGs	Number of covered CpG	Ratio of covered CpG sites (%)
1-CpG	11,187,256	80.8%
3-CpG	8,074,684	58.3%
5-CpG	5,262,625	38.0%
7-CpG	3,244,583	23.4%
9-CpG	1,950,011	14.1%
11-CpG	1,162,851	8.4%
15-CpG	420,460	3.0%
21-CpG	105,979	0.8%
